# Early-career research education mentoring: a successful program in NeuroHIV and mental health (TRNAMH)

**DOI:** 10.1515/nipt-2023-0009

**Published:** 2023-05-08

**Authors:** Heather Thomas, Asante R. Kamkwalala, Avindra Nath, Justin McArthur, Valerie Wojna, Bruce Shiramizu, Ned Sacktor, Carlos A. Pardo, Norman Haughey, Janice Clements, Joseph Mankowski, Christine Zink, Joseph Steiner, Martin Pomper, Linda Chang, Beau Ances, Kurt Hauser, Scott Letendre, Monique Stins, Vivek Nerurkar, Shilpa Buch, Tricia Burdo, Leah H. Rubin, Takashi Tsukamoto, Mikhail Pletnikov, Rachel Salas, Charlene Gamaldo, Peter Dziedzic, Amanda M. Brown

**Affiliations:** Department of Neurology, Johns Hopkins University School of Medicine, Baltimore, MD, USA; Department of Neurology, Johns Hopkins University School of Medicine, Baltimore, MD, USA; National Institute of Neurological Disorders and Stroke, National Institutes of Health, Bethesda, MD, USA; Department of Neurology, Johns Hopkins University School of Medicine, Baltimore, MD, USA; Department of Neurology, University of Puerto Rico, San Juan, PR, Puerto Rico; Department of Tropical Medicine, Medical Microbiology and Pharmacology, University of Hawaii at Manoa, Honolulu, HI, USA; Department of Neurology, Johns Hopkins University School of Medicine, Baltimore, MD, USA; Department of Neurology, Johns Hopkins University School of Medicine, Baltimore, MD, USA; Department of Neurology, Johns Hopkins University School of Medicine, Baltimore, MD, USA; Department of Molecular and Comparative Pathobiology, Johns Hopkins University School of Medicine, Baltimore, MD, USA; Department of Molecular and Comparative Pathobiology, Johns Hopkins University School of Medicine, Baltimore, MD, USA; Department of Molecular and Comparative Pathobiology, Johns Hopkins University School of Medicine, Baltimore, MD, USA; Department of Neurology, Johns Hopkins University School of Medicine, Baltimore, MD, USA; Department of Radiology and Radiological Science, Johns Hopkins University School of Medicine, Baltimore, MD, USA; Department of Neurology, Johns Hopkins University School of Medicine, Baltimore, MD, USA; and Department of Diagnostic Radiology and Nuclear Medicine, and Department of Neurology, University of Maryland School of Medicine, Baltimore, MD, USA; Department of Neurology, Washington University in Saint Louis, Saint Louis, MO, USA; Department of Pharmacology and Toxicology, Virginia Commonwealth University, Richmond, VA, USA; Department of Psychiatry, University of California San Diego, San Diego, CA, USA; Department of Neurology, Johns Hopkins University School of Medicine, Baltimore, MD, USA; Department of Tropical Medicine, Medical Microbiology and Pharmacology, University of Hawaii at Manoa, Honolulu, HI, USA; Department of Pharmacology and Experimental Neuroscience, University of Nebraska Medical Center, Omaha, NE, USA; Department of Microbiology, Immunology, and Inflammation, Temple University School of Medicine, Philadelphia, PA, USA; Department of Neurology, Johns Hopkins University School of Medicine, Baltimore, MD, USA; Department of Molecular and Comparative Pathobiology, Johns Hopkins University School of Medicine, Baltimore, MD, USA; and Department of Epidemiology, The Johns Hopkins University, Baltimore, MD, USA; Department of Neurology, Johns Hopkins University School of Medicine, Baltimore, MD, USA; Department of Epidemiology, The Johns Hopkins University, Baltimore, MD, USA; and Department of Physiology and Biophysics, University of Buffalo School of Medicine and Biomedical Sciences, Buffalo, NY, USA; Department of Neurology, Johns Hopkins University School of Medicine, Baltimore, MD, USA; Department of Neurology, Johns Hopkins University School of Medicine, Baltimore, MD, USA; Department of Neurology, Johns Hopkins University School of Medicine, Baltimore, MD, USA; Department of Neurology, Johns Hopkins University School of Medicine, 600 N. Wolfe St, Meyer 6-119, Baltimore, MD 21287, USA

**Keywords:** biomedical workforce, clinical/translational research, diversity, education, HIV/AIDS, mental health, neurology

## Introduction

### 40-years: an evolving landscape of HIV-associated neurological complications

**Figure 1:**
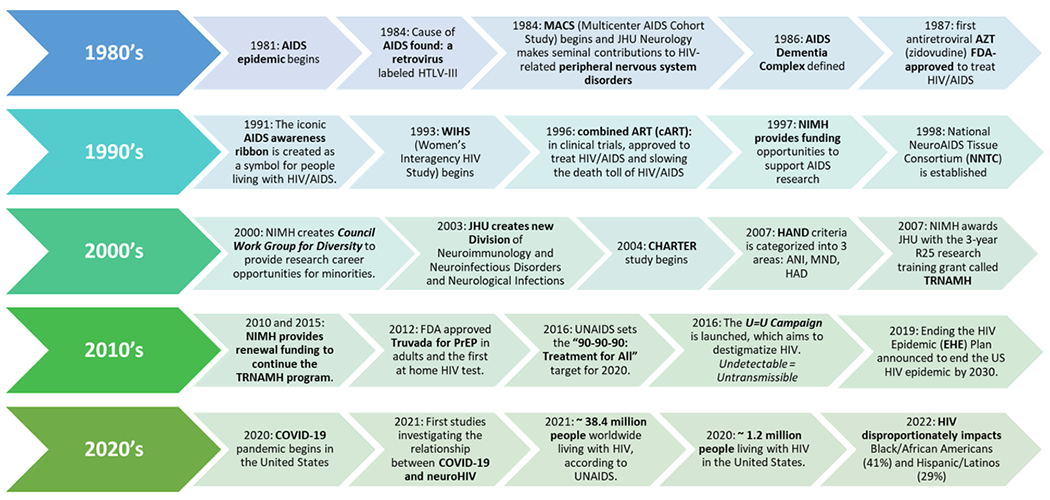
Timeline of the HIV epidemic and notable landmark advances, events and resources related to HIV neurologic complications. HIV-1, human immunodeficiency virus; AIDS, acquired immunodeficiency syndrome; CHARTER, CNS HIV anti-retroviral therapy effects research; HAND, HIV-associated neurocognitive disorders; U=U, undetectable=untransmissible; COVID-19, SARS Co-V 2 coronavirus disease; AZT, azidothymidine.

Understanding the detrimental impacts of HIV infection on the nervous system has been a major research focus since the virus was identified in the late 1980s. Forty years ago, people presenting with clinical symptoms, including severe subcortical cognitive impairment, movement disorders, typically bradykinesia, and gait abnormalities with or without behavioral symptoms, were diagnosed with acquired immune deficiency syndrome (AIDS) dementia complex (ADC). ADC became the first described and clinically used definition of AIDS [1–3] (Figure 1). It was also recognized that HIV affected the spinal cord resulting in a vacuolar myelopathy and peripheral nerve small fiber neuropathy with several scientists at Johns Hopkins contributing to seminal findings to the latter [4–8]. In the decades since the start of the HIV pandemic, scientific innovations in testing, treatment, and management built upon key biologic insights and understanding gained from basic and clinical research, have improved the well-being and life expectancy of millions of people worldwide living with the infection [9–12]. First described in the 1980s through mid-1990s [13–15], understanding of the neurological complications of HIV infection covered a broad range of topics in the life sciences and medical specialties, including neurology/neuroscience, psychiatry/behavior, immunology/infectious disease, and pharmacology.

A consensus about how the neurological impacts of HIV infection of the brain should be identified and classified was reached in 2007 [16]. The designation of “HAND” for “HIV-associated neurologic disorders” was coined and provided a research framework for assessment. A determination of HAND requires the use of a battery of neuropsychological tests, assessments to measure daily living, and clinical evaluations to identify whether and to what extent an individual’s cognitive and motor functions differ from the norm [17, 18]. More recently, the field has moved toward embracing the shift away from the Diagnostic and Statistical Manual of Mental Disorders (DSM) classification system to the Research Domain Criteria (RDoC). RDoC aims to better account for the intricate linkages between disorder presentation and the associated varied biological variables [19]. Saving lives and mitigating the worldwide impact of HIV required simultaneous action and collaboration between clinical and basic science research enterprises [20–23]. Therefore, irrespective of the specific research focus of individual participants, understanding the overall clinical presentation and how people with HIV are identified as suffering from neurological complications was, as described below, a central component of the didactic training component.

Figure 1 shows the timeline of what became an HIV pandemic that spread across countries and borders and evolved into the current ongoing epidemic superimposed with select landmark advances in research, clinical care, and policy. While the United States President’s Plan for AIDS Relief (PEPFAR) funds that support infrastructure and antiviral treatment continue to have a great impact in reducing the spread of the virus, upticks in HIV infection in times of civil unrest, famine, or other disease outbreaks remains a challenge [24, 25]. In the United States, the value of well-characterized cohorts of people with HIV (PWH) such as the NIH-funded Multicenter AIDS Cohort Study (MACS) developed in 1984, and the Women’s Interagency HIV Study (WIHS) initiated in 1993, have contributed significantly to the existing knowledge of HIV neuropathology and clinical outcomes. Today, the two function in a synergistic manner as the “MACS/WIHS Combined Cohort Study” [26–28]. The CNS HIV Antiretroviral Therapy Effects Research (CHARTER), established in 2002 and expanded in 2015, later incorporated the tissue and data management coordinating centers of The National NeuroAIDS Tissue and Consortium (NNTC) and its associated data center [29, 30]. Coincident with further advances in regimens, such as fewer pills involved in combined ART (cART), reduced side effects, and the addition of antiretroviral drugs targeting discrete stages of the HIV replication cycle, came additional improvements in neuropsychiatric outcomes, as well as life-expectancy for people with HIV [17, 31–33]. At Johns Hopkins, a dedicated program to conduct research into neuroHIV, the Northeastern AIDS Dementia Consortium (NEAD) was created in 1998 and marked the founding of the current Division of Neuroimmunology and Neurovirological Infections [34].

In developed countries, advances in HIV research and treatment success have translated into the reality of millions of people aging with HIV. However, without a cure or vaccine, many experience comorbid conditions including cardiovascular and kidney disease, chronic obstructive pulmonary disorder, osteoporosis, frailty, and the continuous threat of lung, breast, colon, and other types of cancers [9, 35–37]. To reflect this data-driven understanding of HIV neuropathogenesis, in 2018, we began to use the term “NeuroHIV or neuroHIV” instead of “NeuroAIDS”. We will use the former notation in this paper hereafter.

### Goal of TRNAMH: nurture a well-trained, resilient, and diverse neuroscience and mental health biomedical research workforce

A properly trained biomedical workforce is required to ensure the USA’s leadership in innovative, high-impact research that benefits the well-being of society. Therefore, educational programming at the graduate level and beyond, which features high-quality training, mentorship, and skill-building in the mechanics of applying for research grants, is essential to advance these goals. Promoting diversity in research has been a stated goal for NIH-funded research institutions since the inception of the Minority Health Initiative in 1992 [38]. Data has continued to accrue which demonstrates that investing in training a more diverse biomedical research workforce, particularly by providing expanded access and support to trainees from URMs and/or those with disabilities, improved matriculation rates into STEM (science, technology, engineering, and mathematics) fields, and leads to more productive and skilled scientific researchers [39, 40]. While the percentage of incoming URM graduate student enrollees in any STEM field has increased on average by 8.4 % between 2011 and 2021 [41], representation in the neurosciences and psychiatric fields at the doctoral level has only increased by about 3.9 % on average in a similar time frame, between 2005 and 2015 [42]. Statistics showed that despite national efforts to encourage and support increased recruitment of diverse, underrepresented minority groups into advanced degree programs and faculty academic research positions in the neurosciences, between 1995 and 2015, doctoral degree recipients in neuroscience comprised only 2.77 % Black/African-Americans, 9.4 % Asian/Pacific Islanders, and 4.3 % Hispanics [42], while more than 70 % were Whites or Asians [43, 44]. These numbers for the URM neuroscience graduates are far below the representation of these groups in the overall US population or even in the pool of URMs who express motivation and aptitude for a career in STEM but later exit from the pipeline [45]. Many studies established that challenges to retention in STEM for women and URMs are associated with the institutional environments and cultures which are often unwelcoming and disaffirming [46].

The premise on which the TRNAMH program was built is that a highly-qualified pool of diverse talent exists and that if given the proper professional support, retention, and advancement in the field will be effectively promoted. The primary aims of the TRNAMH program were to provide mentorship, didactic training, and research support to early-career doctoral students, post-doctoral trainees, and junior faculty with emphasis on those from racial and ethnic URM groups with basic and clinical research interests in the neuroHIV field. The long-term goals were to strengthen foundational skills and knowledge of the neurological and immunological impacts of HIV infection through additional targeted initiatives that included research ethics, grant writing practice, and mentoring, to further promote career development and research independence. Recent critical analyses of evidence-based practices and an understanding of the competencies required for a well-trained neuroscience workforce lend support for the TRNAMH program’s overall design [47].

## Methods and results

### Component I. Evolution and outcomes of the multidisciplinary didactic course from 2007 to 2019

The training program was originally conceived and developed in 2006 by Dr. Avindra Nath, MD, and co-directed with Dr. Amanda Brown, PhD, with R25 funding from the NIMH (R25 MH080661) [48]. Of note, the program was initially named *Diversity-Related Neuro-AIDS and Mental Health Research* and was changed in subsequent funding periods to its current name: *Translational Research in NeuroHIV and Mental Health (TRNAMH)*. The first cohort of trainees began in August 2007, and the final didactic course ended in December 2019. The course emphasized and encouraged a solid foundation in both basic and clinical aspects of neurology, pharmacology, behavioral sciences, psychiatry, epidemiology, and immunology, selecting candidates with an interest in interdisciplinary research into the detection, treatment, and understanding of the various neurobiological and functional outcomes of HIV infection and HAND. Originally, the program was conceived as an institutional collaboration between faculty at the Johns Hopkins School of Medicine, the University of Hawaii at Manoa, and the University of Puerto Rico, Medical Sciences Campus. As the program grew in popularity, additional support and voluntary contributions of time and effort from faculty lecturers and mentors at many more partnering institutions were added and included the: University of California San Diego, University of Nebraska, Temple University, Virginia Commonwealth University, Ponce School of Medicine, Universidad del Caribe, University of Honolulu, Lehigh University, Boston University, Washington University, Drexel University, Hampton University, University of Kentucky, and Georgetown University.

While most program participants possessed a degree of knowledge in the NeuroHIV field, a small percentage was always new by transitioning to graduate school or through an interdisciplinary research collaboration. In this context, the didactic portion of the program always began with a historical clinical and epidemiologic overview followed by a discussion of the latest research findings in neuroHIV. In this regard, and given the availability of the human brain and other organ tissues and body fluids available through the NNTC, CHARTER, ACTG, and NEAD, a complete understanding of these resources is critical for the neuroHIV researcher. At its inception, the coursework was designed to first cover the basic virology and neuropathogenesis of HIV and continue through an examination of the translational and clinical aspects of neuroHIV research and how HAND is treated. Coursework also included key topics on the role of race and ethnicity, cultural sensitivity, the importance of translatability in data collection tools for different populations, and ethics in research.

The course had several unique forward-thinking aspects that contributed to its success. Several of the courses were taught by URM faculty. Course discussions addressed access to and equity in clinical trials many years before (the recent increased attention to this topic [44, 49, 50]). The course embraced a webinar format from its inception as its main mode of teaching and communication long before Skype, Zoom, and other virtual platforms were developed. However, as these newer technologies emerged, the course quickly adopted their use. Importantly, this provided expanded access to both trainees and neuroHIV experts from across the continental USA, Hawaii, and Puerto Rico to join in discussions, didactic teaching, and journal clubs at no cost. Thus, the TRNAMH program was a pioneer in overcoming several institutional and logistical challenges of pre-COVID-19 era traditional teaching formats. In this manner, the course provided mentorship to the trainees, helped them develop research projects, provided pilot funds to select individuals on a competitive basis, and developed lasting relationships with the trainees despite being separated over far geographic distances. This type of investment in the careers of the students was unprecedented and represents a model for other teaching courses and institutions.

#### TRNAMH course dissemination and participant selection

From its first year in 2007 until 2019, the course enrolled a total of 610 trainees from local, national, and international institutions. Year to year, TRNAMH accepted between 22 and 75 students per year into the program (except for 2015, no new trainees were accepted while we awaited grant renewal decisions). In the beginning years of the program, participants were notified of the course mainly through program faculty located at Johns Hopkins, Hawaii, and Puerto Rico. By 2014, the number of course participants more than doubled as it gained visibility across the United States and abroad through word-of-mouth and flyers emailed to sites of the Centers for AIDS Research (CFAR). Trainees in the United States or abroad at the graduate, predoctoral, postdoctoral, and early-career, as well as senior investigators new to the field, were eligible to take the course. All selected trainees completed a *Letter of Intent* and sent it to the Program Administrator before starting the course, which included the participant’s name, institution, degree (e.g., MD, PhD, MS, etc.), level (e.g., pre-doc, post-doc, early career faculty, senior-new to the field, or other), name of primary mentor, race/ethnicity, and a brief description (3–5 sentences) of their training experiences, especially in HIV/AIDS, neuroHIV, mental health, etc.

#### Course participant demographics

**Figure 2:**
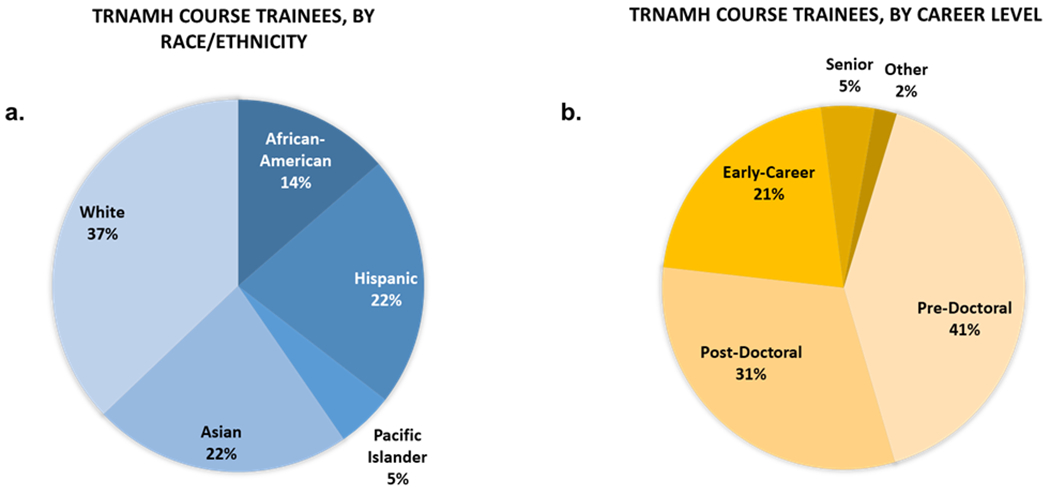
Total racial/ethnic representation (a) and career level (b) of TRNAMH trainees 2007–2019. URM includes the following racial and ethnic groups shown to be underrepresented in biomedical research: Black/African Americans (Black/AA), Hispanics or Latinos, American Indians or Alaska Natives, Native Hawaiians, and other Pacific Islanders.” https://diversity.nih.gov/about-us/population-underrepresented.

Trainees in our program who identified as Asian were predominantly of Indian or Chinese ethnicity. Among our Black/African-American trainees, all of our cohort participants were African-American. We did not have any trainees who identified as Native American/Alaskan Native. While there were significant differences in the number of participants from each racial/ethnic group, over the 12 years, 41 % of trainees came from URM groups (Tables 1–3, Figure 2a). One-way ANOVA analysis of the 5 racial groupings represented in our cohort showed that the proportion of trainees enrolled from each of the defined racial groups (determined by self-report) varied significantly by group from year to year (F (4,55)=11.56, p<0.0001). While the enrollment of those from Asian and Pacific Islander backgrounds was stable, that of Black/African-Americans generally rose each year, and that of Hispanics which began with robust participation that held steady for seven cycles fell off sharply in the later years (Table 1 Graph). The latter can be partially attributed to the detrimental impacts on research infrastructure in Puerto Rico from Hurricane Maria in 2017. The proportion of trainees varied at different career stages with 41 % pre-doctoral, 31 % post-doctoral, 21 % early-career (e.g., research associate, assistant professor), 5 % professor, and 2 % non-degree (other) (Figure 2b).

As part of the core of the program, all participants completed 12 weekly formal didactic lectures. Topics of instruction were administered by experts in their respective fields and included the clinical epidemiology of HIV in the nervous system, neuroimaging, neuropathology of HIV, *in vitro* and *in vivo* disease models, genetics, cognitive and clinical neurological assessment, grant funding advice, as well as the role that racial and ethnic disparities play in study design, ethics, access to care, and representation in the medical and biological sciences (see TRNAMH Course topics, Table 2). Trainees concurrently enrolled at Johns Hopkins University could enroll in the course for 1.5 academic credits with advance notice. Remote trainees could register to receive academic course credits at their home institutions if the courses were approved or they could audit the course for free. NIH grant funding for this program also allowed for tuition remission for those seeking academic credits and who qualified as a trainee from a URM group. The majority of participants chose to audit the course instead of receiving college credit.

Between 2007 and 2009, the 12-week course was offered via webcast videoconferencing, which was at the time considered state-of-the-art technology. Feedback from trainees in these years suggested that while convenient for the different time zones, the webcast format provided limited interactive value with their fellow students and course faculty due to system limitations, glitching, and difficulty in setting up the system using a combination of computers and phone lines. In response to these suggestions, TRNAMH began testing and adopting the use of other interactive web-conferencing platforms, including Adobe Connect and Zoom, which were adopted in 2018 for use in 2019. The platform proved easy to use for the administrators and students in the course, allowing the program to successfully connect with a large cohort of students from across the US and internationally. The format allowed students from anywhere in the country to actively engage in live discussions with program faculty and students in the course and review recorded lectures asynchronously.

As the program continued to expand, additional course topics were added to the didactic curriculum to reflect emerging science, changes in the field, and feedback collected through summative surveys. By 2014, the program transitioned to a 16-week didactic course by including lectures on drugs of abuse, rodent models, sleep comorbidities with app development, and stress-related cognitive issues in PWH. By 2019, the program not only increased the number of faculty and topics due to demand, but the program also expanded even further by reaching trainees from over 30 universities, not only from across the United States, but beyond the US borders to Africa, Asia, Latin America, and Australia.

### Component II. Mentored research scholar experiences and training

A second key goal for the TRNAMH training program was to attract highly qualified, innovative early-career scientists and encourage retention of these researchers in the field of neuroHIV by forging long-lasting connections between trainees and established professionals conducting research in the field. To encourage this, the program had a mentored Research Scholar Initiative. Trainees worked with their home and chosen Johns Hopkins faculty mentor (s) to develop a short (up to 3-month) research project, shadow experience or collect data. The trainee aimed to generate pilot data for future collaborations and grant applications and grow their professional research network in the neuroHIV field. To facilitate a structured and methodical plan for this project, trainees completed a series of guided questions with their TRNAMH mentors, to determine the scope of their project, scientific hypotheses, and specific aims for their progress, including short- and long-term goals for their participation. This aspect of the program was greatly under-utilized despite program coverage of travel and housing costs. Feedback from course participants suggests that it was difficult for many early career trainees to be away from their home institutions due to family obligations and other restrictions, both financial and logistical.

### Component III. Competitive pilot grant funds

Pilot grant funding is pivotal and central to the advancement of early-career investigators as independent researchers, particularly for those who identify as underrepresented racial and gender minorities [51, 52]. Trainees who had taken the didactic course were eligible to apply for a one-year pilot grant in the amount of $20,000 awarded annually on a competitive basis. At the end of the didactic course, the pilot award announcement was sent to all course attendees inviting them to submit a competitive pilot award application.

#### Pilot award application and selection process

To simulate the competitive NIH grant submission process and prepare them for future grant applications, trainees competing for a pilot award were required to submit a 6-page R21 style grant, which included specific aims, a research strategy, pilot data (if available), budget, and biosketches of the principal investigator and other key personnel, as required by the NIH. Highly qualified trainees in the United States who had taken the didactic course were eligible to apply for a $20,000 grant to fund their innovative research projects. Pilot applications were reviewed and scored by the TRNAMH’s Review Committee, which included the Program Director, Co-Director, and the Executive Committee. Each member reviewed and scored the applications on a scale of 1–10 (*1–2: outstanding; 2–3: excellent; 3–4: very good; 4–5: good; 5–6: acceptable; >6: unacceptable*). Criteria for evaluation included innovation, experimental approach, mentoring plan, and expected outcomes. Selected applications were also reviewed by the designated NIH Program Officer for approval of funding before experiments could begin.

In the first year of the pilot grant program, TRNAMH awarded 4 trainees with research funding and has since awarded a total of 37 pilot grants between 2007 and 2019. Table 4, located in the Appendix, provides a full list of the innovative pilot grants by funding year and project title. Seventeen (17) pilot awardees out of 37 (49 %) subsequently received NIH grant funding. In the first iteration of the grant, the majority of candidates for the pilot grant program were identified as an underrepresented minority (URMs) in the sciences (Black/African American, Hispanic, Native American, Native Hawaiian/Pacific Islander). Out of the total pilot awardees, 26 (70 %) were women.

**Figure 3:**
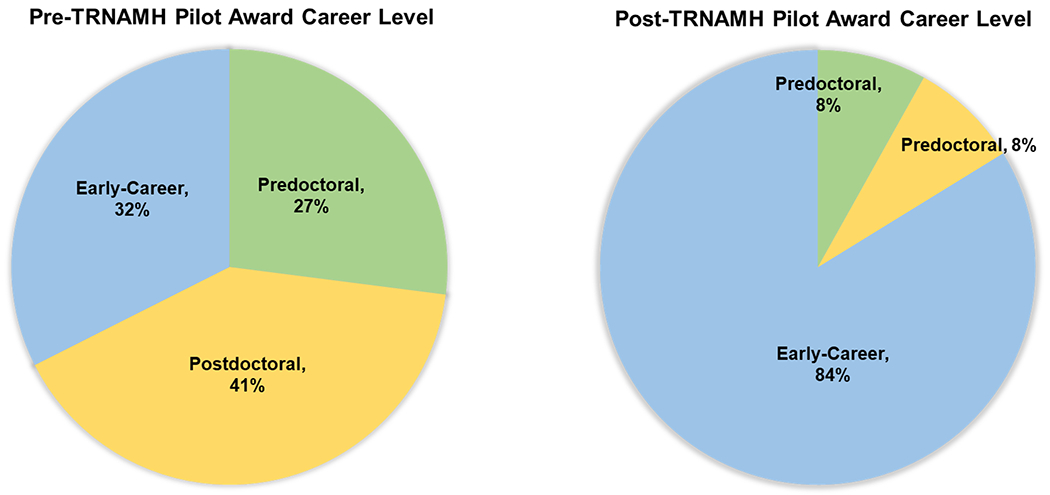
Comparison of career levels before and after receiving a TRNAMH pilot grant.

Due to the program’s very popular pilot grant initiative, renewal eligibility was expanded beyond URMs while maintaining a requirement that the research aligns with the program’s focus on neuroHIV and/or racial disparities affecting biomedical outcomes of the comorbid condition. Successful grant awardees have advanced the goals of this program, with 86 % (32/37) continuing independent research in neuroHIV and associated comorbidities, or mental health-related research (Table 3). The 14 % of awardees who did not continue in academic research are active in clinical, industry, or government-related careers. Of the pilot awardees who are continuing in independent research careers, at the time of surveying in 2021–2022, 84 % are now at the early-career level (Academia, Industry, Government), 8 % are postdoctoral, and 8 % are at the predoctoral level (Figure 3, Table 4).

## Discussion

### Mentorship and collaboration

**Figure 4:**
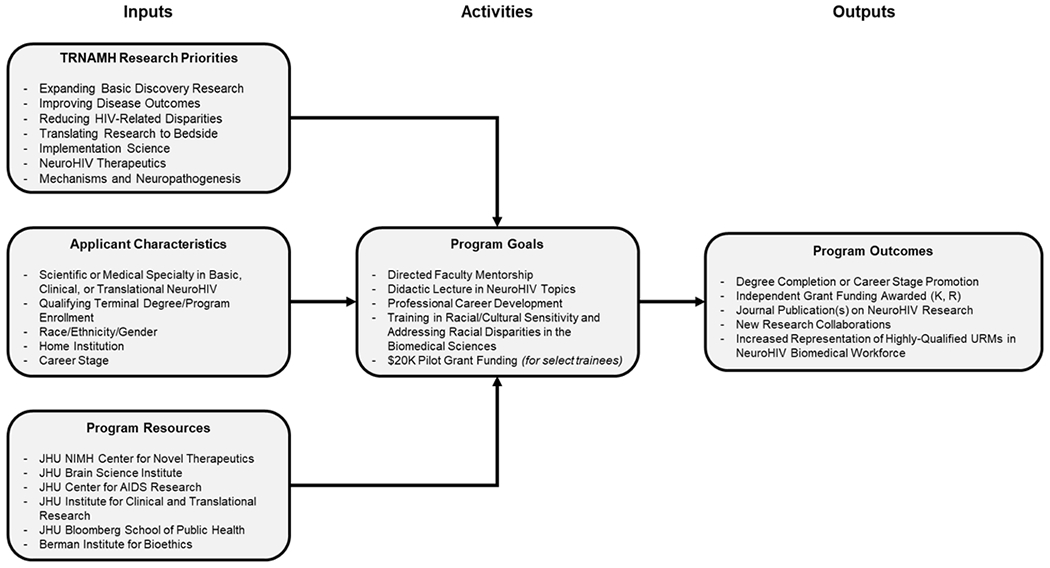
Logic diagram for TRNAMH program. Illustrates the resources, goals, and criteria for program participation, and intended outcomes for course participants.

Providing a strong foundation in the pathophysiology of HIV, its complications in PWH, and the development of treatment strategies, coupled with mentored research and funding mechanisms for new and early-stage investigators were essential components of the model for our program. Especially important for trainees from underrepresented backgrounds is a program where they can be directly mentored and provided with opportunities for career and scientific development. TRNAMH’s network of neuroHIV experts provided the participants with an opportunity to meet, discuss, and build collaborations that may otherwise not have been made without this type of training program. Those who participated in the Research Scholar component were given even more opportunities through hands-on, in-person research experiences. An additional activity of the program in the beginning years included a networking dinner at the Society for Neuroscience conference to facilitate connections between trainees and program faculty. The overall logic diagram of the resources, qualifications, methodology, and desired outcomes of the program is exhibited in Figure 4.

### Ongoing considerations for the TRNAMH course: lessons learned

In the rapidly evolving disciplines of neuroHIV, the TRNAMH program has been successful in supporting highly qualified early-career students and scientists from underrepresented minorities, a key factor in improving the effectiveness and innovation of research in this field. As previously discussed, with a disease like HIV infection that disproportionately affects non-white communities worldwide, having a more diverse cohort of neuroHIV researchers would be a monumental step towards more equitable, valid, and generalizable research [39]. Previous reports showed that the lack of diversity in the biomedical workforce relative to the general population may be a key factor that continues to contribute to health disparities and inequalities [53]. The goal of reducing these racial and ethnic disparities in our trainee pool and subsequently retaining them in the neuroHIV field were key features of the TRNAMH program.

Diversity and inclusion initiatives began in earnest within biomedical research training programs; however, translating the outcomes of these programs into demonstrable changes in the makeup of the research workforce remains a challenge. While some improvements were seen at the graduate student level (over the past 10 years, there have been reported increases in the proportion of female, underrepresented minorities, and/or disabled graduate applicants, students, and post-docs [41, 42]), the overall diversity of junior and senior faculty, professors, and major grant awardees has not changed commensurately. This represents a multi-layered barrier at the early-career level for these diverse students to succeed, a combination of inadequate opportunities for continued research support, high-level training, or other interpersonal factors that lead these individuals to leave the biomedical workforce or academic tenure track. Training programs like TRNAMH seek to resolve these barriers, providing additional opportunities that these trainees may not otherwise be given, to overcome institutional (structural racism, unconscious bias) and historical gaps (inequity, geography) that often limit professional progress.

The success of the TRNAMH program was also evident from feedback received from the trainees. The trainees reported significant value in the experience and a high likelihood of recommending this program to colleagues within neuroHIV research. In particular, the trainees who were awarded pilot grant funding have gone on to be successful, innovative researchers in their fields even at an early career stage, with the majority continuing to do neuroHIV research to this day. TRNAMH made headway in addressing several barriers that were identified as potential reasons for the lag in more minority representation in the biomedical research fields by providing a scientific network, hands-on research experiences, and pilot grant funding, which also provided tailored mentorship. In line with our program’s goal to develop a neuroHIV research workforce from the ground up that is more representative of the racial diversity of the general population, our outcome data thus far shows a promisingly diverse enrollment in the TRNAMH course (Figure 2a), as well as the majority of our trainees entering the program (72 %) at the predoctoral or postdoctoral level (Figure 2b). This small investment in the continued engagement of the trainees can have long-lasting and tangible benefits.

Capitalizing on the web-based format of the program played a significant role in expanding our reach to institutions, faculty, and trainees worldwide. Virtual technology improvements allowed for the program to continue despite trainees learning at a distance, from their home institutions. However, feedback from both trainees and faculty mentors acknowledged some challenges to participation in this program without face-to-face involvement. The improvements in our virtual platforms over the years, as well as the opportunities for on-site research training for students in the program, and pilot grant funding, helped to offset institutional infrastructure limitations that are encountered at their institutions, also providing access to techniques and methodology that might not have been available except with collaborations fostered by Johns Hopkins and our extensive collaborative network of investigators. Adopting technology early in the process to enhance communication across the continent and across time zones is another key feature and foresight of the developers of this course that contributed to its success.

Though the improvements in online platforms used by TRANMH have mostly benefitted and streamlined access, communication, and interaction for the trainees, virtual participation in the TRNAMH program did face some difficulty accommodating trainees who had other academic obligations at their home institutions. Scheduling time with mentors, lab meetings, and consistent check-ins, despite the time-zone differences were some of the unanticipated difficulties of not bringing all trainees locally to Baltimore, although we believe that we have made and will continue to make significant improvements in these areas over time. After our grant renewal in 2015, we altered the admissions criteria for the program, allowing non-URM trainees to participate in the course, provided that they were still conducting research in the neuroHIV field and/or addressing racial/ethnic disparities in the biomedical sciences. However, the subsequent decline in the proportion of URM trainees in these more recent years may reflect a need for a renewed effort to continue to recruit consistently higher proportions of these URM trainees who may also face the additional challenge of navigating post-graduate training amid the COVID-19 pandemic, which has been shown to disproportionately and negatively affect people and students of color [54–57]. Whether these challenges are financial, interpersonal, or logistical, remain to be seen, but addressing these additional barriers will certainly be an important focus as we continue to determine how best to ensure equitable access to high-quality biomedical neuroscience and HIV research training for the future years of our program.

TRNAMH’s yearly feedback surveys helped to determine whether participants felt that they had gotten value out of the course, and ways that the program could be improved. These surveys were extremely useful, providing insight into the motivations of our trainees, and confirming our hope that the course is achieving the set goals of the program for the trainees it is intended to serve. Boxs 1 & 2 provide a snapshot of trainee responses to survey questions related to personal value and ways to improve the program.

### Addressing ongoing challenges to biomedical workforce diversity

In 2019, neuroHIV researcher Dr. David Stoff identified 4 major challenges to the goal of successfully diversifying the workforce of researchers in our field [39]: (1) inadequate understanding of the unique workforce dynamics that contribute to or detract from feelings of belonging for URMs [58, 59]; (2) poor retention of URM trainees and scientists or “leaky” professional advancement pipelines that discourage these individuals from progressing [45, 46, 60]; (3) inefficient integration of R25 funding mechanisms into the infrastructure of academic research departments to support trainees [61, 62], and (4) qualities of the institution’s mentor climate that do not sufficiently support the needs of the URM trainees [63–65]. Our program has made significant progress addressing these points, focusing on training the cohort to understand the impact of their work in the context of racial and ethnic disparities that exist not only in the incidence and impact of HIV but the workforce that studies it; offering additional funding and mentorship to overcome pipeline leaks that may have otherwise led some of these professionals to leave the field; and allowing our URM trainees the opportunity to learn from and be mentored by faculty professionals who are also themselves, URMs. Though the R25 funding that supported this program has not yet overcome Dr. Stoff’s challenge 3, we believe the other successes are evidence that more progress is not just feasible, but necessary. In particular, the pilot funding that our program was able to provide was significant: as indicated in Box 2, at least one of our cohort members confirmed that additional funding, by raising the pilot grant award from $20–50 K, would have been even more impactful. Prior studies showed that early career stage funding plays a key role in fostering successful scientific careers, particularly in members of these URM groups, which in turn is likely to contribute to overcoming many of the barriers that remain for successful initiatives for diverse academic faculty [51, 52]. With the addition of enhanced mentored training, which we provided in our TRNAMH program, career development progress for URM scientists in these fields is also expected to accelerate [66].

In conclusion, the Translational Research in NeuroHIV and Mental Health program achieved 12 years of outstanding progress toward improving the breadth, depth, and diversity of the researchers engaged in neuroHIV research. The development of this program addressed key areas of improvement in this branch of the biomedical sciences. The unique aspects of this program as discussed above represent a model for others to follow. All the same, we intend to continue addressing the feedback received over the last 12 years from our participants, institutions, and trainees to continue to enrich mentorship opportunities, expand the curriculum, and fund highly innovative research geared towards minimizing disparities and improving access to opportunities and resources towards a better understanding of neuroHIV.

## Figures and Tables

**Table 1 and Graph:** Demographic characteristics of TRNAMH course trainees by year, 2007–2019.

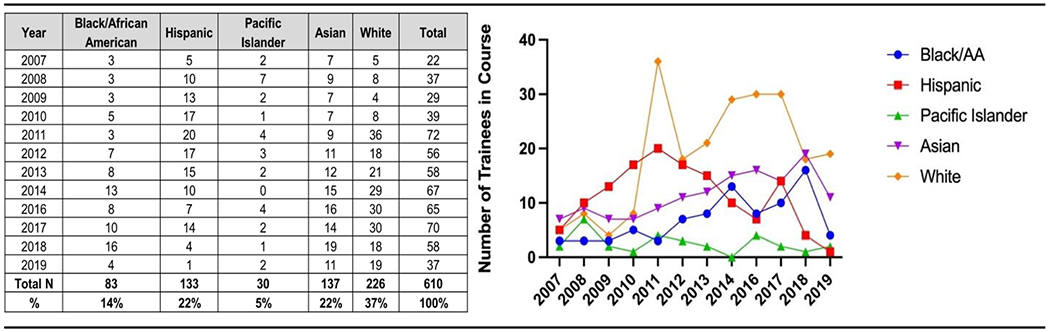

The TRNAMH program did not run in 2015, as grant fund renewal was pending. The graph shows the data in Tables 1–4 in a longitudinal manner to show trends in enrollment.

**Table 2:** Didactic syllabus for translational research in NeuroHIV and mental health (TRNAMH) course, 2007–2019. The table shows the faculty lead discussants and their associated academic institutions. Darker shading indicates new courses that were added in 2014 to make a 16-week course from 2014 to 2019.

Course Topics: 12-weeks (2007-2013) and 16-weeks (2014-2019)	Faculty Instructor(s):	Institution(s):
1. Course Overview and Clinical Epidemiology of HAND	Amanda Brown, PhD Ned Sacktor, MD	Johns Hopkins University
2. Intro to Retroviruses and Pathology of NeuroHIV	Vivek Nerurkar, PhD Carlos Pardo, MD	University of Hawaii, Manoa Johns Hopkins University
3. Cultural Sensitivity in Neuropsychological Scales for HAND / Ethics in NeuroHIV Research	Valerie Wojna, MD Bruce Shiramizu, MD	University of Puerto Rico, Medical Sciences Campus University of Hawaii, Manoa
4. Neuropathogenics of HIV Infection and IRIS	Avindra Nath, MD	National Institute of Neurological Disorders and Stroke
5. Viral Genetics and Cellular Reservoirs	Janice Clements, PhD Joseph Mankowski, DVM, PhD	Johns Hopkins University
6. Drugs of Abuse in HAND	Shilpa Buch, PhD Kurt Hauser, PhD	University of Nebraska Virginia Commonwealth University
7. Surrogate and Host Genetic Markers	Norman Haughey, PhD	Johns Hopkins University
8. Blood-Brain Barrier and HIV	Monique Stins, PhD	Johns Hopkins University
9. CNS Penetration, HIV-1 Replication and Combination Anti-Retroviral Therapies	Scott Letendre, MD	University of California, San Diego
10. Rodent Models and Behavioral Assessment	Mikhail Pletnikov, MD, PhD Amanda Brown, PhD	Johns Hopkins University
11. Drug Development for HAND	Joseph Steiner, PhD Takashi Tsukamoto, PhD	National Institute of Neurological Disorders and Stroke Johns Hopkins University
12. Imaging Techniques in NeuroHIV	Beau Ances, MD, PhD Linda Chiang, MD	Washington University in St. Louis University of Maryland School of Medicine
13 Clinical Research Techniques and How to Get and Stay Funded	Justin McArthur, MBBS, MPH	Johns Hopkins University
14. Cardiovascular Disease in HIV	Tricia Burdo, PhD	Temple University
15. Sleep and HIV/App Development	Charlene Gamaldo, MD Rachel Salas, MD Peter Dziedzic, MS	Johns Hopkins University
16. The Wear and Tear of Stress on Cognition in HIV	Leah Rubin, PhD, MPH	Johns Hopkins University

**Table 3:** TRNAMH pilot grant awardee characteristics and outcomes by race. Racial identity was determined via self-report.

	Total	Black	Hispanic	Pacific Islander	Asian	White
Number of Pilot Awardees	37	9	8	3	6	11
Number of Women	26	8	4	3	2	9
**Pre-TRNAMH Career Level**						
Pre-Doctoral	10	3	1	1	1	4
Post-Doctoral	15	3	2	1	4	5
Early Career	12	3	5	1	1	2
**Post-TRNAMH Career Level**						
Pre-Doctoral	3	1	0	0	0	2
Post-Doctoral	3	0	0	1	0	2
Early Career	31	8	8	2	6	7
**Post-TRNAMH Career and Funding**						
Academia	28	4	6	3	5	10
Clinical	3	3	0	0	0	0
Industry	5	1	2	0	1	1
Government	1	1	0	0	0	0
Ongoing NeuroHIV Research	32	5	8	3	6	10
NIH Grants Funded	26	5	3	2	9	7
**Publications**						
Total	1066	354	138	38	195	341
Total Post-Pilot Award	721	242	103	36	98	242
First Author	167	55	22	7	19	64
Middle Author	444	133	68	29	73	141
Last Author	110	54	13	0	6	37

## Box 1: Selected Trainee Reflections on Benefits of TRNAMH Course Participation

**Table:**

**Question 1: Since your participation in the program, could you comment on the long term impacts it has had on your career progression?**		
“I got to see how the network of HIV/AIDS research is like which gives me a good feeling for how large the network is.” “I have learned a lot from the program. The process of planning, applying grants and reporting helped prepare me into the next phase of my research career. It is a very valuable resource ful of mentorship and resources that still help me today.” “It inspired and encouraged me for getting a career development award on HIV psychiatric comorbidities.”	“I have learned a lot form the program. The processor planning, applying grants and reporting helped prepare me into the next phase of my research career. It is a very valuable resource full of mentorship and resources that still help me today.”	“It helped me to gain funding in the neuro-HlV field that has led to career advancement.” “I am able to manage patients suffering from HIV/AIDS with competency. As i progressed in my careerto more mentoring of junior researchers, the program continued to provide a tool to help my faculty and trainees build their knowledge and networks, this program has had a marked impact on my career and that of many others.” “The program introduced me to experts in a variety of NeuroHIV and HAND related areas of research. I was able to expand my professional network, which was critical.”
	“This program helped solidify my interest in NeuroAIDS and helped me appreciate the complexity of the disease.”	
	“This didactic program provide a new insight about new cutting edge research in neurological disorder and mental health issues in people living with HIV/AIDS.”	
**Question 2: If such a course would be offered in the future would you recommend it to others?**		Response rate: 98%* – “Extremely Likely”
*Selected responses received in 2021.*		

*: On a 1-100% Scale

## Box 2: Selected Trainee Recommendations for Future Improvements to TRANMH Course

**Table:**

**Question 3: Reflecting on your past participation in the TRNAMH course, what would improve it or make it better for future participants?**		
“I would suggest to include more group projects.”	“I think you should think about increasing the funding from 20K to 50K.” “Experts coming in and giving a lecture is an old-fashioned siloed learning modality. The level of interactivity is limited by participants asking questions and many participants might struggle to speak up. Alternatively, the experts should create a hybrid interactive learning approach where they share their latest knowledge and require a select group of participants to also share their research findings on that topic, with the hope to create a brainstorm session of sorts.” “Make it more clinical based as opposed to research based.”	“I recommend to add a course on leadership about team building and crisis management.”
“The timing is difficult with the different time zones. Also the online meeting software can be updated. But this was when I took the course in 2018.”		“Allowing the research grant opportunities to fund international projects.”
“As the course has evolved, it may be good to make archived versions available to current participants and current versions available to prior participants.”		“It’s my keen and humble suggestion to make it better for future participants is some critical thinking work exercise related to each lecture.”
*Selected responses received in 2021.*		

## Appendix

**Table 4:** Award years and project titles of Innovative pilot awards funded by the TRNAMH Program from 2007 to 2019. The most recent projects were completed by 2021.

Award Year	Pilot Awardee	Project Title
**2019-2021**	Sanjay Kumar, PhD, MS	“HIV-1 entry into neuronal/glial cells and the underlying mechanism(s) of exosome-dynamics in cocaine abuse”
**2019-2021**	Palsamy Periyasamy, PhD (+)	“HIV Tat-mediated astrocyte activation involves NLRP6-mediated pyroptosis: Implications for neuroinflammation in HAND”
**2019-2021**	Susmita Sil, PhD (+)	“Molecular mechanisms involved in Alzheimer’s like comorbidity of HAND”
**2019-2020**	Claire Lyons, DVM	“Effect of Social Stress on Dopaminergic and Serotonergic Pathways in the CNS of SIV Infected Macaques”
**2019-2020**	Danielle Clements PhD	“Investigation of Co-receptor Usage in a Myanmar Pediatric HIV Cohort Using Next Generating Sequencing and Implications for Neurocognitive Outcomes”
**2018-2019**	Audrey Knight, PhD	Dysregulation of central nervous system glial immune responses in a cART--suppressed SIV/macaque model of chronic HIV CNS disease
**2018-2019**	Bethany A. Reinecke, PhD	Bivalent Ligand Approach to Explore MOR-CXCR4 Crosstalk in Opioid Enhanced NeuroAIDS
**2018-2019**	Ashutosh Tripathi, PhD	Combinatorial effects of HIV & cART in mediating microglial activation via dysregulated autophagy: Implications for neuroinflammation underlying HAND
**2018-2019**	Bianca Cotto, PhD	Combined Effects of HIV-1 Tat and Cocaine on Astrocyte Metabolic Functions and the Impact on to Rapid Aging in the CNS
**2017-2018**	Mario Bianchet, PhD (+)	Regulation of HIV-1 Tat proteostasis as a latency switch and neuroprotective strategy
**2017-2018**	Kristen Hollinger, PhD	Neutral Sphingomyelinase 2 Inhibition for the Treatment of HIV-Associated Neurocognitive Disorders
**2017-2018**	Crystal Leibrand, PharmD, PhD	Structural and Functional Consequences of HIV-1 Viral Proteins and Morphine Exposure at the Blood-Brain Barrier
**2017-2018**	Xiaolei Zhu, MD, PhD (+)	Treating HIV-associated neurocognitive disorder (HAND) using a traditional Chinese medicine ingredient: Repairing gut microbiota dysbiosis-induced sustained inflammation via inhibition of nonTLR-mediated inflammatory signaling
**2016-2017**	Dionna Williams, PhD (+)	“Antiretroviral Therapy Efficacy in the Central Nervous System”
**2016-2017**	Minglei Guo, PhD (+)	“Studies Related to the Effects of Drugs of Abuse and/or HIV on CNS Dysfunction”
**2013-2014**	Raissa Menendez-Delmestre, PhD	Interplay of HIV-associated neurocognitive disorders with obesity
**2013-2014**	Kimberly Smith, MD	A streamlined assessment program to promote engagement in care for traditionally marginalized populations following an HIV diagnosis
**2013-2014**	Yoelvis Garcia Mesa, PhD	Ligand-activated nuclear receptors regulate HIV latency in microglial cells
**2012-2013**	Bryan Smith, MD (+)	The effects of aerobic exercise on cognitive performance for individuals with HIV-associated cognitive disorder
**2012-2013**	April D. Thames, PhD (+)	Proinflammatory genotypes and susceptibility to HIV-associated neurocognitive disorder in African-Americans
**2012-2013**	Rosa J. Rodriguez PhD, MPH, MS	Validation and use of the spatial navigation memory island test in evaluation of HIV-associated cognitive disorder
**2012-2013**	Sarah E. Beck, MD (+)	Immune selection of SIV in the CNS: costs and consequences
**2012-2013**	David Alvarez-Carbonell, PhD, MBA	The fractalkine and ErbB receptors as regulators of HIV latency in infected microglia cells: implications for HIV-associated neurocognitive disorders
**2012-2013**	Yamil Gerena, PhD (+)	Soluble insulin receptor, lipidomic dysregulation, and insulin resistance in HIV+ women with HAND
**2012-2013**	Jennifer Lyons, MD	Remote assessment of HAND in Cape Coast, Ghana: a pilot study
**2011-2012**	Kelly Metcalf Pate, DVM, PhD (+)	The role of thrombopoietin in the pathogenesis of SIV-associated CNS disease
**2010-2011**	Nicholas Kenney, MD (+)	Detection of Cripto-1 in murine cortical brain tissue
**2011-2012**	Crystal Bethel-Brown, PhD, MBA	HIV-1 Tat and alcohol-mediated disruption of brain endothelium: role of platelet-derived growth factor-BB (PDGF-BB)
**2011-2012**	Valeria Avdoshina, MD, PhD (+)	Contribution of TrkB promoter mutation to HIV-associated depression
**2009-2011**	Valerie Toodle, PhD	Neuroprotective and neurorestorative effects of fluconazole against HIV proteins and mitochondrial toxins
**2009-2011**	Marilou Andres, PhD (+)	*APOE* expression in the CSF of patients with HIV-1 infection
**2009-2011**	Crystal Watkins, MD, PhD	Imaging immune mechanisms of depression in older HIV patients
**2009-2011**	Jose W. Rodriguez, MD (+)	Neuroprotective effect of tobacco cembranoid: Therapeutic implications for HIV-associated neurocognitive disorders
**2008-2010**	Summer Acevedo, PhD	Association between 14-3-3e, cytokines, and HIV-associated neurocognitive disorders in women
**2008-2010**	Charlene Gamaldo, MD	The relationship between HIV-associated neuropathy and insomnia
**2008-2010**	Juliana Perez-Laspuir, PhD (+)	Neuroprotective candidates for HIV-associated neurocognitive disorders
**2008-2009**	Melissa Agsalda, PhD (+)	HIV DNA in CNS compartments about HIV-associated neurocognitive disorders

(+) Indicates pilot awardee received subsequent NIH grant funding since receiving the TRNAMH pilot award.

## References

[1] Navia BA, Jordan BD, Price RW. The AIDS dementia complex: I. Clinical features. Ann Neurol 1986;19:517–24. 3729308 10.1002/ana.410190602

[2] Portegies P, de Gans J, Lange JM, Derix MM, Speelman H, Bakker M, Declining incidence of AIDS dementia complex after the introduction of zidovudine treatment. BMJ 1989;299:819–21. 2510843 10.1136/bmj.299.6703.819 PMC1837716

[3] Grant I, Heaton RK. Human immunodeficiency virus type 1 (HIV-1) and the brain. J Consult Clin Psychol 1990;58:22–30. 2181001 10.1037//0022-006x.58.1.22

[4] Kaku M, Simpson DM. Neuromuscular complications of HIV infection. Handb Clin Neurol 2018;152:201–12. 29604977 10.1016/B978-0-444-63849-6.00016-5

[5] Kamin SS, Petito CK. Idiopathic myelopathies with white matter vacuolation in non-acquired immunodeficiency syndrome patients. Hum Pathol 1991;22:816–24. 1869265 10.1016/0046-8177(91)90211-7

[6] Cornblath DR, McArthur JC. Predominantly sensory neuropathy in patients with AIDS and AIDS-related complex. Neurology 1988;38:794–6. 2834669 10.1212/wnl.38.5.794

[7] McArthur JC, Yiannoutsos C, Simpson DM, Adornato BT, Singer EJ, Hollander H, A phase II trial of nerve growth factor for sensory neuropathy associated with HIV infection. AIDS Clinical Trials Group Team 291. Neurology 2000;54:1080–8. 10720278 10.1212/wnl.54.5.1080

[8] Pardo CA, McArthur JC, Griffin JW. HIV neuropathy: insights in the pathology of HIV peripheral nerve disease. J Peripher Nerv Syst 2001;6:21–7. 11293804 10.1046/j.1529-8027.2001.006001021.x

[9] High KP, Brennan-Ing M, Clifford DB, Cohen MH, Currier J, Deeks SG, HIV and aging: state of knowledge and areas of critical need for research. A report to the NIH Office of AIDS Research by the HIV and Aging Working Group. J Acquir Immune Defic Syndr 2012;60(1 Suppl):S1–18. 22688010 10.1097/QAI.0b013e31825a3668 PMC3413877

[10] Lohse N, Hansen ABE, Gerstoft J, Obel N. Improved survival in HIV-infected persons: consequences and perspectives. J Antimicrob Chemother 2007;60:461–3. 17609196 10.1093/jac/dkm241

[11] TATCC. Life expectancy of individuals on combination antiretroviral therapy in high-income countries: a collaborative analysis of 14 cohort studies. Lancet 2008;372:293–9. 18657708 10.1016/S0140-6736(08)61113-7 PMC3130543

[12] May MT, Ingle SM. Life expectancy of HIV-positive adults: a review. Sex Health 2011;8:526–33. 22127039 10.1071/SH11046

[13] Grant I, Atkinson JH, Hesselink JR, Kennedy CJ, Richman DD, Spector SA, Evidence for early central nervous system involvement in the acquired immunodeficiency syndrome (AIDS) and another human immunodeficiency virus (HIV) infections. Studies with neuropsychologic testing and magnetic resonance imaging. Ann Intern Med 1987;107:828–36. 3688675 10.7326/0003-4819-107-6-828

[14] Goethe KE, Mitchell JE, Marshall DW, Brey RL, Cahill WT, Leger GD, Neuropsychological and neurological function of human immunodeficiency virus seropositive asymptomatic individuals. Arch Neurol 1989;46:129–33. 2916951 10.1001/archneur.1989.00520380029008

[15] Miller EN, Seines OA, McArthur JC, Satz P, Becker JT, Cohen BA, Neuropsychological performance in HIV-1-infected homosexual men: the multicenter AIDS cohort study (MACS). Neurology 1990;40:197–203. 2405289 10.1212/wnl.40.2.197

[16] Antinori A, Arendt G, Becker JT, Brew BJ, Byrd DA, Cherner M, Updated research nosology for HIV-associated neurocognitive disorders. Neurology 2007;69:1789–99. 17914061 10.1212/01.WNL.0000287431.88658.8b PMC4472366

[17] Chan P, Brew BJ. HIV associated neurocognitive disorders in the modern antiviral treatment era: prevalence, characteristics, biomarkers, and effects of treatment. Curr HIV AIDS Rep 2014;11:317–24. 24966139 10.1007/s11904-014-0221-0

[18] Grant I. Neurocognitive disturbances in HIV. Int Rev Psychiatry 2008;20:33–47. 18240061 10.1080/09540260701877894

[19] Sanislow CA, Morris SE, Cuthbert BN, Pacheco J. Development and environment in the national Institute of mental health (NIMH) research Domain criteria. J Psychopathol Clin Sci 2022;131:653–9. 35901394 10.1037/abn0000768

[20] Munoz A, Schrager LK, Bacellar H, Speizer I, Vermund SH, Detels R, Trends in the incidence of outcomes defining acquired immunodeficiency syndrome (AIDS) in the Multicenter AIDS Cohort Study: 1985-1991. Am J Epidemiol 1993;137:423–38. 8096356 10.1093/oxfordjournals.aje.a116691

[21] Roffman RA, Stephen RS, Curtin L, Gordon JR, Craver JN, Stern M, Relapse prevention as an interventive model for HIV risk reduction in gay and bisexual men. AIDS Educ Prev 1998;10:1–18. 9505095

[22] Ulett KB, Willig JH, Lin HY, Routman JS, Abroms S, Allison J, The therapeutic implications of timely linkage and early retention in HIV care. AIDS Patient Care STDS 2009;23:41–9. 19055408 10.1089/apc.2008.0132 PMC2733237

[23] Stein DS, Lyles R, Graham N, Tassoni C, Margolick J, Phair J, Predicting clinical progression or death in subjects with early-stage human immunodeficiency virus (HIV) infection: a comparative analysis of quantification of HIV RNA, soluble tumor necrosis factor type II receptors, neopterin, and beta2-microglobulin. Multicenter AIDS Cohort Study. J Infect Dis 1997;176:1161–7. 9359714 10.1086/514108

[24] Mann M, Lurie MN, Kimaiyo S, Kantor R. Effects of political conflict-induced treatment interruptions on HIV drug resistance. AIDS Rev 2013;15:15–24. 23449225 PMC3774601

[25] Gisselquist D. Impact of long-term civil disorders and wars on the trajectory of HIV epidemics in sub-Saharan Africa. SAHARA J 2004;1:114–27. 17601017 10.1080/17290376.2004.9724834

[26] Barkan SE, Melnick SL, Preston-Martin S, Weber K, Kalish LA, Miotti P, The women’s interagency HIV study. WIHS collaborative study group. Epidemiology 1998;9:117–25. 9504278

[27] D’Souza G, Bhondoekhan F, Benning L, Margolick JB, Adedimeji AA, Adimora AA, Characteristics of the MACS/WIHS combined cohort study: opportunities for research on aging with HIV in the longest US observational study of HIV. Am J Epidemiol 2021;190:1457–75. 33675224 10.1093/aje/kwab050 PMC8484936

[28] Detels R, Jacobson L, Margolick J, Martinez-Maza O, Muñoz A, Phair J, The multicenter AIDS cohort study, 1983 to … Publ Health 2012;126:196–8. 10.1016/j.puhe.2011.11.013 PMC3324261 22206985

[29] Letendre SL, Marquie-Beck J, Ellis RJ, Woods SP, Best B, Clifford DB, The role of cohort studies in drug development: clinical evidence of antiviral activity of serotonin reuptake inhibitors and HMG-CoA reductase inhibitors in the central nervous system. J Neuroimmune Pharmacol 2007;2:120–7. 18040835 10.1007/s11481-006-9054-y

[30] Morgello S, Gelman BB, Kozlowski PB, Vinters HV, Masliah E, Cornford M, The National NeuroAIDS Tissue Consortium: a new paradigm in brain banking with an emphasis on infectious disease. Neuropathol Appl Neurobiol 2001;27:326–35. 11532163 10.1046/j.0305-1846.2001.00334.x

[31] Del Palacio M, Alvarez S, Munoz-Fernandez MA. HIV-1 infection and neurocognitive impairment in the current era. Rev Med Virol 2012;22:33–45. 21990255 10.1002/rmv.711

[32] Kranick SM, Nath A. Neurologic complications of HIV-1 infection and its treatment in the era of antiretroviral therapy. Continuum 2012;18:1319–37. 23221843 10.1212/01.CON.0000423849.24900.ec PMC3760534

[33] Mothobi NZ, Brew BJ. Neurocognitive dysfunction in the highly active antiretroviral therapy era. Curr Opin Infect Dis 2012;25:4–9. 22156897 10.1097/QCO.0b013e32834ef586

[34] Sacktor N, McDermott MP, Marder K, Schifitto G, Selnes OA, McArthur JC, HIV-associated cognitive impairment before and after the advent of combination therapy. J Neurovirol 2002;8:136–42. 11935465 10.1080/13550280290049615

[35] Stoff DM, Goodkin K, Jeste D, Marquine M. Redefining aging in HIV infection using phenotypes. Curr HIV AIDS Rep 2017;14:184–99. 28933001 10.1007/s11904-017-0364-x PMC5614907

[36] Vance DE, Mugavero M, Willig J, Raper JL, Saag MS. Aging with HIV: a cross-sectional study of comorbidity prevalence and clinical characteristics across decades of life. J Assoc Nurses AIDS Care 2011;22:17–25. 20471864 10.1016/j.jana.2010.04.002

[37] Dausey DJ, Desai RA. Psychiatric comorbidity and the prevalence of HIV infection in a sample of patients in treatment for substance abuse. J Nerv Ment Dis 2003;191:10–7. 12544594 10.1097/00005053-200301000-00003

[38] NIMHD. History. National Institute on Minority Health and Health Disparities; 2010. Available from: https://www.nimhd.nih.gov/about/overview/history/ [Accessed 2023].

[39] Stoff DM. Enhancing diversity and productivity of the HIV behavioral research workforce through research education mentoring programs. AIDS Behav 2019;23:2889–97. 31129748 10.1007/s10461-019-02520-w PMC6789045

[40] Stoff DM, Cargill VA. Building a more diverse workforce in HIV/AIDS research: the time has come. AIDS Behav 2016;20:222–30. 27484058 10.1007/s10461-016-1501-z

[41] Zhou E. Graduate enrollment and degrees: 2011-221. Washington, DC: C.o.G. Schools; 2022.

[42] Ramos RL, Alviña K, Martinez LR. Diversity of graduates from bachelor’s, master’s and doctoral degree neuroscience programs in the United States. J Undergrad Neurosci Educ 2017;16: A6–13. 29371835 PMC5777839

[43] Jones HP, Thorpe RJ Jr., Vishwanatha JK. The National Institute of Neurological Disorders and Stroke’s efforts on diversifying the neuroscience research workforce. J Neurosci Res 2022;100:1545–50. 34085300 10.1002/jnr.24852

[44] Oh SS, Galanter J, Thakur N, Pino-Yanes M, Barcelo NE, White MJ, Diversity in clinical and biomedical research: a promise yet to Be fulfilled. PLoS Med 2015;12:e1001918. 26671224 10.1371/journal.pmed.1001918 PMC4679830

[45] Wong B, Chiu YLT, Murray ÓM, Horsburgh J. End of the road? The career intentions of underrepresented STEM students in higher education. Int J STEM Educ 2022;9:51. 35966573 10.1186/s40594-022-00366-8 PMC9362640

[46] Roche R, Manzi J, Baker S, Ndubuizu T. Underrepresented minority students and identification of obstacles to a career in medicine. Clin Teach 2021;18:186–90. 33258266 10.1111/tct.13312

[47] Institute of Medicine (IOM). Developing a 21st century neuroscience workforce: workshop summary, Norris SMP, editor, Washington, DC: The National Academies Press; 2015:128 p. 25905158

[48] Brown A, Shiramizu B, Nath A, Wojna V. Translational research in NeuroAIDS: a neuroimmune pharmacology-related course. J Neuroimmune Pharmacol 2011;6:80–8. 20496178 10.1007/s11481-010-9222-y PMC3155799

[49] Rotsides JM, Moses LE, Malloy KM, Brenner C, Fayson SM, Brown DJ, Disparities in access to translational research. Curr Probl Cancer 2022;46:100894. 35989105 10.1016/j.currproblcancer.2022.100894

[50] Fryer CS, Passmore SR, Maietta RC, Petruzzelli J, Casper E, Brown NA, The symbolic value and limitations of racial concordance in minority research engagement. Qual Health Res 2016;26:830–41. 25769299 10.1177/1049732315575708 PMC4658313

[51] Heggeness ML, Ginther DK, Larenas MI, Carter-Johnson FD. The impact of postdoctoral fellowships on a future independent career in federally funded biomedical research. Cambridge, MA: National Bureau of Economic Research; 2018.

[52] Hiatt RA, Carrasco YP, Paciorek AL, Kaplan L, Cox MB, Crespo CJ, Enhancing grant-writing expertise in BUILD institutions: building infrastructure leading to diversity. PLoS One 2022;17:e0274100. 36137156 10.1371/journal.pone.0274100 PMC9499285

[53] Sullivan LW. Association of academic health centers, missing persons: minorities in the health professions, a report of the Sullivan Commission on diversity in the healthcare workforce. Atlanta, GA: Sullivan Commission; 2004:201 p.

[54] Barber PH, Shapiro C, Jacobs MS, Avilez L, Brenner KI, Cabral C, Disparities in remote learning faced by first-generation and underrepresented minority students during COVID-19: insights and opportunities from a remote research experience. J Microbiol Biol Educ 2021;22:ev22i1.2457. 10.1128/jmbe.v22i1.2457 PMC8046656 33884088

[55] Boncyk M, Froese S, Ambikapathi R, Verissimo C, Matangi E, Ruiz Y, Social disparities and food environment determinants of food insecurity among graduate students in the United States during the COVID-19 pandemic. Curr Dev Nutr 2021;5:107.

[56] Schad A, Layton R, Ragland D, Cook J. Uncovering the compounding effects of COVID-19 and racism on mental health disparities among biomedical PhD and MD students. eLife 2021. 10.1101/2021.04.29.21251164.

[57] Walsh BA, Woodliff TA, Lucero J, Harvey S, Burnham MM, Bowser TL, Historically underrepresented graduate students’ experiences during the COVID-19 pandemic. Fam Relat 2021;70:955–72. 34548724 10.1111/fare.12574 PMC8444674

[58] Kaundinya T. Facilitating identity compatibility in mentorships: implications for diversity in medicine. J Med Educ Curric Dev 2021;8:23821205211006412. 10.1177/23821205211006412 PMC8728776 35005239

[59] O’Brien LT, Garcia DM, Blodorn A, Adams G, Hammer E, Gravelin C. An educational intervention to improve women’s academic STEM outcomes: divergent effects on well-represented vs. underrepresented minority women. Cult Divers Ethnic Minor Psychol 2020;26:163–8. 10.1037/cdp0000289 31021140

[60] Avakame EF, October TW, Dixon GL. Antiracism in academic medicine: fixing the leak in the pipeline of Black physicians. ATS Sch 2021;2:193–201. 34409414 10.34197/ats-scholar.2020-0133PS PMC8357067

[61] Flores G, Mendoza FS, DeBaun MR, Fuentes-Afflick E, Jones VF, Mendoza JA, Keys to academic success for underrepresented minority young investigators: recommendations from the research in academic pediatrics initiative on diversity (RAPID) national advisory committee. IntJ Equity Health 2019; 18:93. 31215424 10.1186/s12939-019-0995-1 PMC6582500

[62] Ofili EO, Tchounwou PB, Fernandez-Repollet E, Yanagihara R, Akintobi TH, Lee JE, The research centers in minority institutions (RCMI) translational research network: building and sustaining capacity for multi-site basic biomedical, clinical and behavioral research. Ethn Dis 2019;29:135–44. 30906162 10.18865/ed.29.S1.135 PMC6428183

[63] Bath EP, Brown K, Harris C, Guerrero A, Kozman D, Flippen CC, For us by us: instituting mentorship models that credit minoritized medical faculty expertise and lived experience. Front Med 2022;9:966193. 10.3389/fmed.2022.966193 PMC9634999 36341236

[64] Javier D, Stinson K, Zavala M, Ahmed T, Vishwanatha JK. NRMNet: building a national resource for mentorship, networking and professional development to enhance diversity. Ethn Dis 2021;31:469–80. 34295135 10.18865/ed.31.3.469 PMC8288472

[65] Williams LB, Surratt HL, King VL, Kern PA. The Disparities Researchers Equalizing Access for Minorities (DREAM) Scholars program: career development for underrepresented health equity researchers. Clin Transl Sci 2021;5:e170. 10.1017/cts.2021.845 PMC8532178 34733546

[66] Rice TK, Jeffe DB, Boyington JE, Jobe JB, Davila-Roman VG, Gonzalez JE, Mentored training to increase diversity among faculty in the biomedical sciences: the NHLBI summer Institute programs to increase diversity (SIPID) and the programs to increase diversity among individuals engaged in health-related research (PRIDE). Ethn Dis 2017;27:249–56. 28811736 10.18865/ed.27.3.249 PMC5517143

